# Mechanical Properties and Water Absorption Capacity of Hybrid GFRP Composites

**DOI:** 10.3390/polym14071394

**Published:** 2022-03-29

**Authors:** Wan Hamidon Wan Badaruzzaman, Noaman Mohammed Ridha Dabbagh, Kushairi Mohd Salleh, Esri Nasrullah Saharuddin, Nur Fashiha Mat Radzi, Mohd Amir Ashraff Azham, Shahrul Faizi Abdullah Sani, Sarani Zakaria

**Affiliations:** 1 Department of Civil Engineering, Universiti Kebangsaan Malaysia (UKM), Bangi 43600, Selangor, Malaysia; esri95@gmail.com (E.N.S.); fashiharadzi@gmail.com (N.F.M.R.); amirashraff822@gmail.com (M.A.A.A.); shahrulfaizi.sf@gmail.com (S.F.A.S.); 2 Department of Applied Physics, Faculty of Science & Technology, Universiti Kebangsaan Malaysia (UKM), Bangi 43600, Selangor, Malaysia; szakaria@ukm.edu.my

**Keywords:** hybrid GFRP composites, kenaf fibre, coconut fibre, fly ash, nano-silica, calcium carbonate, water absorption capacity, mechanical properties

## Abstract

Hybrid glass fibre reinforced polymer (GFRP) composites have been used for decades in various engineering applications. However, it has a drawback with its application in marine/flood environments due to a lack of water resistance and frail mechanical stability. Floods have been considered one of the most periodic hazards that could hit urban areas, due to climate change. The present paper aims to address this gap and to investigate the mechanical properties (tensile, compressive, and flexural strength) and water absorption capacity of hybrid GFRP composite comprising woven E-glass fabric and epoxy resin, various reinforcing materials (kenaf and coconut fibres), and various filler materials (fly ash, nano-silica, and calcium carbonate (CaCO_3_). The composites with 30 wt.% GFRP, 50 wt.% resin, 15 wt.% fly ash, 5 wt.% CaCO_3_, 10 wt.% GFRP, 60 wt.% resin, and 30 wt.% fly ash showed the lowest water absorption property of 0.45%. The results revealed that the GFRP composite reinforced kenaf fibres with nano-silica, fly ash, and CaCO_3_ improved the water absorption resistance. At the same time, GFRP reinforced the coconut fibres with fly ash, and kenaf fibres with CaCO_3_ showed no favourable impact on water absorption. The identification of a hybrid GFRP composite with various reinforcing materials and fillers would assist future developments with a more compatible, enhanced, and reliable water-resistant composite, specifically for structural applications in flood-prone areas.

## 1. Introduction

Hybrid glass fibre reinforced polymer (GFRP) composite is a composite material that combines two types of natural fibres, or natural and synthetic fibres, with one matrix or two polymer blends [1]. Therefore, the hybrid composite could be tailored to fit the required properties in real applications and has gained popularity in many industries like aerospace, automotive, and construction [1]. Before applying hybrid GFRP in structures that will be exposed to flood risk, additional parameters are required for further consideration. The composite material proposed must comply with the Federal Emergency Management Agency (FEMA) guidelines for flood situations. The materials chosen should be able to withstand the dry–wet cycle and resist direct contact with flood water for at least 72 h without leaving any significant damage [2]. According to FEMA, water absorption properties are the most important aspects that should be seriously considered. Reddy et al. [3] investigated the absorption rate of the GFRP composite at different times. The results showed a slight increase for the first 1 h, a very slow increase between 1 to 2.5 h, and finally a drastic increase between 2.5 to 4 h. The total percentage of water absorption after 4 h was 11.7%. A study on seawater absorption conducted by Chakraverty et al. [4] for 365 days showed that the percentage moisture gain for seawater could reach 1.5%.

Raja et al. [5] studied the mechanical properties of a fly ash impregnated E-glass GFRP composite. By comparing different composite compositions, the results showed that the 10 wt.% fly ash fillers in the FRP (70 wt.% resin with 20 wt.% fibre) possessed optimum mechanical properties due to their excellent adhesion. However, for a higher wt.% of fly ash, the mechanical properties showed degradation due to voids around the fly ash particles, which caused poor interfacial adhesion. Therefore, Raja et al. [5] suggested that utilising a smaller grain size of fly ash filler could have solved the problem.

Sravani et al. [6] investigated the effect of CaCO_3_ and aluminium oxide (Al_2_O_3_) composition as fillers on the mechanical properties of GFRP composites. A decrease in tensile and flexural strengths was observed at different filler percentages. However, the impact resistance and hardness of the composite showed a significant improvement when CaCO_3_ was introduced as a filler. Furthermore, an increasing trend of water absorption capacity was recorded when the filler percentage was increased.

Sapiai et el. [7] studied the influence of nano-silica (5, 13, and 25 wt.%) on the tensile and flexural properties of a woven glass/unidirectional kenaf hybrid composite. An improvement in the mechanical properties was recorded in the presence of nano-silica. Based on the scanning electron microscopy (SEM), the nano-silica was distributed homogenously in the epoxy matrix, which enhanced the adhesion bonding between the composition of the hybrid composite. Furthermore, the composition of treated silica and non-treated kenaf and hybrid composite systems significantly improved the flexural performance.

Coconut fibres are considered to be eco-friendly, durable, weather-resistant, relatively waterproof, and chemically modified. Natural fibre reinforced polymer-composites (NFCs) are reported to have high corrosion, high impact resistance, high stress to weight ratio, low maintenance requirements, and non-conductive properties [8]. According to Lekube et al. [9], two methods can be used to measure the porosity of the composite, which are a very important factors; the first was mainly based on the measurements of the absolute density, while the second was mainly based on image analysis of micro-computed tomography scans. The water absorption capacity of a coconut hybrid GFRP composite was investigated by Bhagat et al. [10]. The results showed that the water absorption of this composite increased significantly when the fibre length and immersion time increased.

An experimental and numerical investigation was conducted by Quino et al. [11] to study the effect of water absorption on the mechanical properties of GFRPs composites. With a high water absorption capacity, the stiffness, and tensile and shear strengths of the GFRPs composites were gradually reduced. This was attributed to the weakness of the fibre-matrix and interlaminar interfaces. However, the ductility of the GFRPs composites showed an increasing trend. Furthermore, the re-drying of fully saturated GFRPs composites showed partial recovery of the mechanical properties.

Chellamuthu and Vasanthanathan [12] investigated the tensile performance under the marine environment of a new, improved performance GFRP composite consisting of glass fibre and polyethylene terephthalate (PET). The introduction of PET in the composition revealed a superior tensile strength with less water absorption.

Al-Sabagh et al. [13] examined the potential of utilisation of carbon nanofibers (CNFs) and multi-wall carbon nanotubes (MWCNTs) for reducing the water absorption capacity and its effects on the mechanical properties, as well as monitoring the propagation of moisture damage in GFRP composites. After exposure to seawater, the results showed a significant decrease in the glass transition temperature and storage modulus. This is attributed to the degradation in the epoxy network by reducing the epoxy crosslinking. CNFs coupons showed an improve performance compared to the MWCNT counterpart.

Based on the previous studied, the utilisation of GFRP composites in infrastructure projects (marine/flood environment) has recently gained significant interest. Hence, this study investigated the effect of using hybrid nanofiller materials such as CaCO_3_, fly ash, and nano-silica in the hybrid reinforced fibre of glass fibre with coconut fibre or kenaf fibre blended in the epoxy resin polymer matrix on the mechanical and water absorption properties.

## 2. Materials and Methods

### 2.1. Materials

Seven different materials were used to fabricate the hybrid GFRP composite. Woven glass fabric and polyester resin were adopted as the main GFRP composite body, and natural fibre of kenaf and coconut coir fibres served as the reinforcing materials. The designation of hybrid GFRP composites (17 groups in total with 141 replicates) is shown in Table 1. In the composite designation, the letter G refers to GFRP, R refers to resin, F refers to fly ash, CaCO_3_ relates to calcium carbonate, K refers to kenaf fibres, N refers to nano-silica, and C refers to coconut coir fibres. The numbers refer to the percentage of each composition. For example, 40G-50R-9K-1N-GFRP represents the GFRP composite composition of 40% woven GFRP, 50% resin, 9% kenaf fibres, and 1% nano-silica.

A Woven E-glass fabric mat (woven rowing mat) and epoxy resin with hardener were supplied by Cenco Sains Sdn. Bhd., Selangor, Malaysia and Akzo Nobel, Selangor, Malaysia, respectively, and were used without any further treatment. The epoxy resin was mixed with hardener at a weight ratio of 2:1.

Kenaf bast fibres (bio retting process) were supplied by LKTN, Kelantan, Malaysia. The kenaf bast fibres were alkaline treated with 5% sodium hydroxide. The fibres were air-dried for 2 days until the moisture content reached less than 10%, and they were stored in a closed tight bag. While the shredded coconut coir fibres were supplied by a local supplier and were used without any surface treatment. Both kenaf bast fibres and coconut coir fibres were arranged in a unidirectional orientation for composite making.

Fly ash in powder form was supplied by an ACE greencement venture (M) Sdn. Bhd., Selangor, Malaysia and the source was from the Jimah Power Plant, Negeri Sembilan, Malaysia. The fly ash powder of various sizes was used as received. Nano silica in liquid form (Cembinder 8) and CaCO_3_ (analytical grade) were supplied by R&M Chemical, Selangor, Malaysia and were used as received.

### 2.2. Fabrication of Hybrid GFRP Composite

A hand layup technique was used for all of the specimen fabrication. A mould with a size of 200 mm × 200 mm × 10 mm was prepared, and releasing agent, polyvinyl alcohol, was coated on the inner base and wall of the mould. The matrix material was prepared from the epoxy resin and hardener at a weight ratio of 2:1. The fly ash/nano-silica/CaCO_3_ fillers were added to the resin and were stirred thoroughly before pouring into the mould. The resin mixture was applied inside the mould surface, and the glass fibre mat and kenaf fibres/coconut fibres were arranged unidirectionally inside the mould. Then, the resin matrix was poured onto the fibres. A mild steel roller was used to ensure no air bubbles were left in the sample. The sample was cured at room temperature for 24 h. After the curing process, the samples were removed from the mould and cut into different sizes according to the ISO standards and test specifications.

### 2.3. Mechanical Testing and Water Absorption Capacity

The 30 kN capacity Universal Testing Machine (Instron, Norwood, MA, USA), 2000 kN Compression Machine (Unitest Scientific Sdn. Bhd., Petaling Jaya, Malaysia), and Flexural Machine (Via Salvo D’Acquisto, Milan, Italy) were used to measure the tensile, compression, and flexural properties of the hybrid GFRP composite according to ISO (572, 604, and 178) standardisation, respectively. The loading rate was 10 mm/min for the tensile test, 1 mm/min for the compression test, and 2 mm/min for the flexural test. Furthermore, the ISO 62 standard for moisture absorption of plastic was utilized to measure the water absorption capacity. The weights of the samples before and after being dipped in distilled water were then measured to the nearest 0.01 g after 1 min of moisture removal on the surface of the samples. The percentage of water absorbed was obtained by the weight gained in percentage compared to the initial weight of the samples.

## 3. Results and Discussion

### 3.1. Tensile Strength

**Figure 1:**
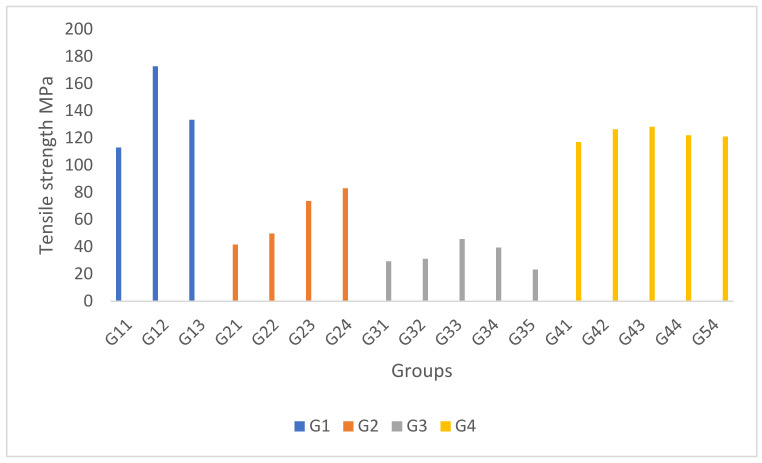
Tensile strength of the hybrid GFRP composites.

The results for G1 show that with the increase in CaCO_3_ from 5% to 10% and the decrease of fly ash from 15% to 10%, the tensile strength substantially increased but started decreasing when mixed with 15% CaCO_3_ and 5% fly ash, as illustrated in Figure 1. This increase was due to the effective synergistic effect between CaCO_3_ and fly ash at a similar percentage, which strengthened the adhesion. A corresponding finding was reported by [14,15], where the results from the microscopic imaging examination showed that with the addition of CaCO_3_, a favourable roughness was found in the fibre surfaces, leading to better bonding between the fibres in the matrix. Furthermore, a significant stress transfer and fewer crack formations were reported in Raja et al. [5] due to the better interaction between the fly ash and the matrix. However, as the percentage of CaCO_3_ and fly ash was dissimilar, the composite matrix was unbalanced, where the immiscibility led to a weakening effect. In G2, the results showed that the tensile strength increased gradually with the increase of the nano-silica composition and the decrease in kenaf fibres composition. A higher nano-silica content increased the surface area for efficient physical interactions with other substituents. The enhancement of physical interactions promoted adhesion between the matrix and reinforcing materials, thus resulting in a better tensile property. In G3, it was noted that the tensile strength relatively increased with the fly ash content for the lowest percentage of coconut fibres. A higher coconut fibre content promoted the formation of voids; meanwhile, a higher fly ash content filled the voids created by the coconut fibres. When the voids were filled with fly ash fibres, the stress transfer was more even, leading to better tensile properties [5]. Bhagat et al. [10] found a gradual increase in the tensile strength associated with a relative increase in volume fraction of voids when the coconut fibre length was 15 mm. Nevertheless, when the hybrid GFRP composite was supplemented with fly ash or coconut fibres only, the tensile strength of G35 (10G-60R-30F-GFRP) was lower than G34 (10G-60R-30C-GFRP). The lower tensile value was due to the low fly ash dispersibility. Subsequently, there were no voids to fill, initiating agglomeration and creating weak points on the hybrid GFRP composite. In G4, the best tensile strength was obtained from the 15% kenaf fibre composition and at 5% CaCO_3_. A reduction in tensile strength values was recorded when the kenaf fibres or the CaCO_3_ composition was 0%. Table 2 shows the tensile strength comparisons between different hybrid GFRP composites.

### 3.2. Compressive Strength

**Figure 2:**
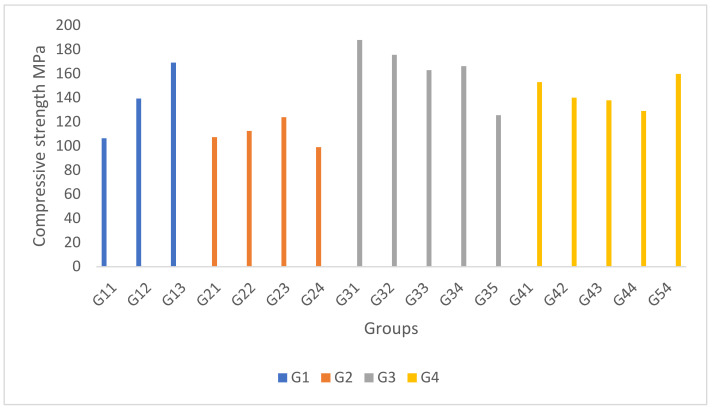
Compressive strength of the hybrid GFRP composites.

The compressive strength results obtained from G1 showed an increasing trend when the composition of the filler CaCO_3_ was increased with the association of decreasing in the composition of the fly ash filler. CaCO_3_ had better compressibility than the fly ash filler. Meanwhile, in G2, an increasing trend of compressive strength was shown to be associated with a reduction of kenaf fibres and the addition of nano-silica. Therefore, nano-silica tends to enhance the physical interaction and adhesion of the composite. However, when the nano-silica content was 1% (9% kenaf fibres), the compressive strength was substantially reduced, even lower than the lowest nano-silica content of 0.25% (9.75% kenaf fibres). The sudden reduction showed the importance of kenaf fibres for complementing the compressive stress. If the loaded kenaf fibres were too low, the stress transfer could be concentrated and not evenly distributed, even at a higher nano-silica content. In G3, the 15% coconut fibre and fly ash composition of G31 showed that the composite experienced optimum compressive strength. A decrease in compressive strength was observed with increased fly ash due to a weak interfacial bond, making the composite more brittle. Similar to the tensile strength, the compressive strength of G35 was lower than G34, consistent with the given deductions. In G4, the compressive strength showed a decreasing trend with an increase in kenaf fibre percentage, with reduced CaCO_3_. The finding was in line with the G1 samples, where higher CaCO_3_ instilled a better compressibility and vice versa. The results for compressive strength are shown in Figure 2 and Table 2.

### 3.3. Flexural Strength

**Figure 3:**
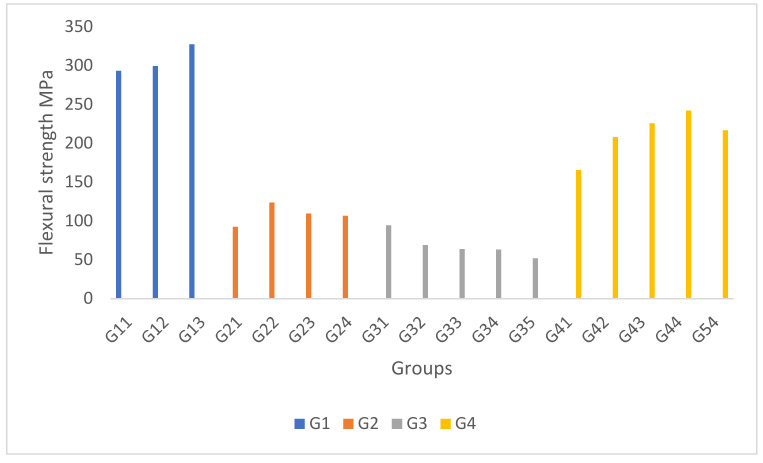
Flexural strength of the hybrid GFRP composites.

The flexural strength of G1 is also depicted in Figure 3. An increasing trend associated with increasing the percentage of the CaCO_3_ and decreasing the fly ash can be observed. This behaviour was due to the high interfacial bond between the composite compositions and the uniformly distributed resin along with the GFRP fibres. The trend was similar as for the compressive strength, but not the tensile strength. Usually, a synonymous result between flexural and tensile strength would be obtained if the blending materials were homogenous. This suggests that inhomogeneity occurred in the hybrid GFRP composites, leading to a local stress concentration during the tensile test. In G2, the result showed that nano-silica substantially impacted the flexural strength. However, as the nano-silica percentage increased (higher than 0.5%), clustering particles of nano-silica fillers weakened the interfacial bond of the matrix. In G3, the results showed that a similar percentage of coconut fibres and fly ash at 15% gave the optimum flexural strength. Further increase of fly ash and reduction of coconut fibres resulted in a low flexural strength, even lower than the lowest fly ash content for the G3 sample. This shows that coconut fibres played an important role in sustaining good flexural strength, as proven with G34 with a higher flexural value than G35. In G4, associated with the increasing percentage of kenaf fibre, the flexural strength showed an increasing trend. Table 2 shows the effect of each composition on the flexural strength.

### 3.4. Water Absorption Capacity

**Figure 4:**
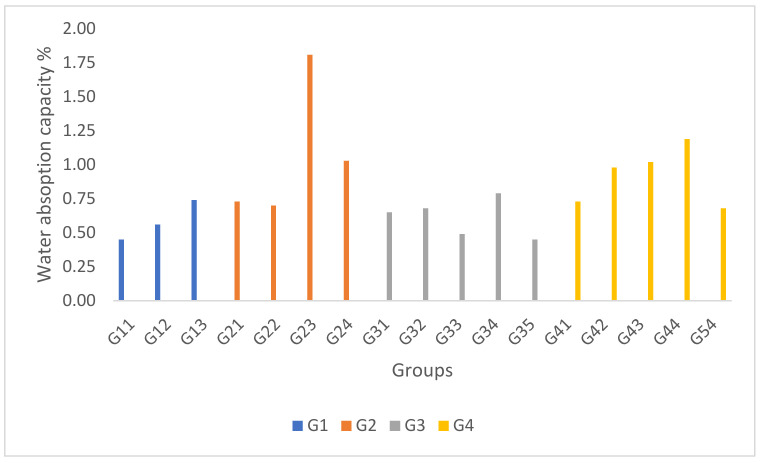
The water absorption capacity of the hybrid GFRP composites.

Water absorption capacity is considered the most pivotal material property in a flood environment. Hence, a lower water absorption capacity is the most favourable as resistance to flooding would be more significant and impervious for real applications. In G1, the results showed that the water absorption capacity increased significantly at lower fly ash and a higher percentage of CaCO_3_, as seen in Figure 4. Based on the mechanical properties results in the G1 samples, the fly ash had no merit for improving the mechanical performance of the hybrid GFRP composite. However, fly ash has been proven to instigate a hydrophobic character for water-resistant applications [16]. Meanwhile, CaCO_3_ caused a significant increment of water absorption value; therefore, it is not suggested to be incorporated in the hybrid GFRP composite. In G2, unlike for the mechanical properties, in the water absorption value, the incorporation of nano-silica and kenaf fibres showed irregular results. Supposedly, water absorbency should be lower at a higher percentage of nano-silica, as nano-silica is a hydrophobic material. Meanwhile, water absorbency should be higher with a higher kenaf fibre content, as kenaf fibre is a hydrophilic material. The irregularity is caused by the inhomogeneity between these two materials. Formations of the void are disproportionate and inconsistent [17,18]. Due to this, a higher water absorption value for G23 was not literally caused by the materials, but was motivated by erratic formations of the voids [19]. Bajuri et al. [18] found similar void formations that caused similar phenomena. The natural fibres like kenaf bast fibre and coconut coir fibre possess a higher hydroxyl group (from polysaccharides chain) and tend to be very hydrophilic. Thus, it resulted in higher swelling properties due to the affinity of moisture and water to form hydrogen bonding with these fibres, increasing the water absorption or water uptake. It could also be concluded that a large portion of water absorbed in the G2 samples was free water, not primary or secondary water [20]. In G3, similar occurrences were spotted as in the G1 sample, as seen in Figure 4. The higher fly ash content interpolated the hydrophobic character, with an opposite hydrophilic character at a higher percentage of coconut fibres. In addition, the G35 sample obtained the lowest absorption among all of the samples, undoubtedly due to its highest fly ash content of 30%. However, the higher fly ash content lowered the mechanical performances of the hybrid GFRP composites. As reported elsewhere [21], filler, especially of a nano size (higher surface area), usually will interrupt the adhesion between resin and reinforced fibre (in this case, woven glass fibre and natural fibres), thus reducing their mechanical strength. The excessive addition of filler (sometimes they tend to agglomerate) may result in the formation of voids, reducing the mechanical strength and allowing for the water cluster area where water was absorbed and remained as free water [5]. Even though lower water absorption is desirable, a balance between mechanical performances and water resistance must be considered. As for G4, a higher CaCO_3_ content did not increase the hybrid composite’s water absorption like in the G1 composites. The major factor for the higher water absorption in the G4 samples was due to the increase in kenaf bast fibres. Therefore, based on the irregular trend shown between the G1 and G4 samples, it can be concluded that CaCO_3_ had no significant effect on the water absorption capacity when mixed with other filler or reinforcements. Nonetheless, it is undeniable that CaCO_3_ showed a considerable impact on the mechanical properties of the hybrid GFRP composite. Table 2 shows the comparisons of the water absorption capacity between different hybrid GFRP composites.

## 4. Techno-Economics Aspects

It is essential to consider the techno-economic aspects of the research. However, this was not one of the objectives of this paper. The developed composite material is intended to be applied in flooding situations as a construction material. Coconut and kenaf fibres were proposed, as these materials are natural fibres that are cheaper and are easily available from the local market. The water absorption resistance of the hybrid GFRP reinforced kenaf fibre composites with fly ash and CaCO_3_ was enhanced. However, the results showed that GFRP reinforced coconut fibres with fly ash and kenaf fibres with CaCO_3_ showed no favourable impact on water absorption. Therefore, the challenge would be to conduct further research to find the best or optimum composition that would finally result in more techno-economic composite materials.

## 5. Conclusions

The compressive and flexural strengths of the composites increased as the content of the CaCO_3_ filler increased. The content of fly ash filler influenced the water absorption of the composite. The higher the fly ash filler, the lower the water absorption. Both fillers influenced the tensile strength, as the highest ultimate strength of the composite had 10% CaCO_3_ and fly ash. Moreover, nano-silica and fly ash fillers have reduced the water absorption rate and compressive strength. Meanwhile, weak interfacial bonding between the resin and fibres that caused the air void trapped in the specimens influenced the mechanical results. Coconut and kenaf fibres are more robust in their tensile and flexural strengths, but the formation of hydrogen bonding with water increased the water absorbency of GFRP composites. On the other hand, calcium carbonate filler helped reduce the water absorption rate and compressive strength. Therefore, the resulting properties of the GFRP composite according to various reinforcing materials and fillers were beneficial for the customisation of flood structural refrainers.

## Figures and Tables

**Table 1:** The designation of hybrid GFRP composites.

Group	Sample	Designation	Composition wt. %						
			Glass Fabric	Resin	Kenaf Fibre	Coconut Fibre	Fly Ash	Nano Silica	CaCO_3_
G1	G11	30G-50R-15F-5CaCO_3_-GFRP	30	50	-	-	15	-	5
	G12	30G-50R-10F-10CaCO_3_-GFRP	30	50	-	-	10	-	10
	G13	30G-50R-5F-15CaCO_3_-GFRP	30	50	-	-	5	-	15
G2	G21	40G-50R-9.75K-0.25N-GFRP	40	50	9.75	-	-	0.25	-
	G22	40G-50R-9.5K-0.5N-GFRP	40	50	9.5	-	-	0.5	-
	G23	40G-50R-9.25K-0.75N-GFRP	40	50	9.25	-	-	0.75	-
	G24	40G-50R-9K-1N-GFRP	40	50	9	-	-	1	-
G3	G31	10G-60R-15C-15F-GFRP	10	60	-	15	15	-	-
	G32	10G-60R-22.5C-7.5F-GFRP	10	60	-	22.5	7.5	-	-
	G33	10G-60R-7.5C-22.5F-GFRP	10	60	-	7.5	22.5	-	-
	G34	10G-60R-30C-GFRP	10	60	-	30	-	-	-
	G35	10G-60R-30F-GFRP	10	60	-	-	30	-	-
G4	G41	20G-60R-5K-15CaCO_3_-GFRP	20	60	5	-	-	-	15
	G42	20G-60R-10K-10CaCO_3_-GFRP	20	60	10	-	-	-	10
	G43	20G-60R-15K-5CaCO_3_-GFRP	20	60	15	-	-	-	5
	G44	20G-60R-20K-GFRP	20	60	20	-	-	-	-
	G45	20G-60R-20CaCO_3_-GFRP	20	60	-	-	-	-	20

G: Glass Fabric, R: Resin, K: Kenaf Fibre, C: Coconut Fibre, F: Fly Ash, N: Nano Silica, GFRP: Glass Fibre Reinforced Polymer.

**Table 2:** Comparative matrix of the tested specimens.

Property	G1	G2	G3	G4
Tensile strength	Tensile strength increased when CaCO_3_ increased and fly ash decreased.	Tensile strength increased when nano silica increased, and kenaf fibre decreased.	Tensile strength increased when fly ash increased, and coconut fibre decreased.	Tensile strength increased when kenaf fibre increased and CaCO_3_ decreased.
Compressive strength	Compressive strength increased when CaCO_3_ increased and fly ash decreased.	Compressive strength increased, nano silica increased, and kenaf fibre decreased.	Compressive strength decreased when fly ash increased, and coconut fibre decreased.	Compressive strength decreased when kenaf fibre increased, and CaCO_3_ decreased.
Flexural strength	Flexural strength increased when CaCO_3_ increased and fly ash decreased.	Flexural strength increased when nano silica increased, and kenaf fibre decreased.	Flexural strength decreased when fly ash increased, and coconut fibre decreased.	Flexural strength increased when kenaf fibre increased and CaCO_3_ decreased.
Water absorption capacity	Water absorption capacity increased when CaCO_3_ increased and fly ash decreased.	Water absorption capacity showed irregular results.	Water absorption capacity increased when fly ash decreased and coconut fibre increased.	Water absorption capacity increased when kenaf fibre increased, and CaCO_3_ decreased.

## Data Availability

Not applicable.
